# Challenges of biomedical research collaboration in India: Perceptions of Indian and international researchers

**DOI:** 10.1371/journal.pone.0305159

**Published:** 2024-06-28

**Authors:** Vaishali Deshmukh, Tanuja Agarwala, Archisman Mohapatra, Sanjiv Kumar, Sushma Acquilla, Manoja K. Das, Rajib Dasgupta, Sanjay Chaturvedi, Subrata Sinha, Sharmila Mukherjee, Mubashir Angolkar, Naveet Wig, Navneet K. Dhamija, Narendra Kumar Arora, on behalf of the INCLEN Leadership Study Group

**Affiliations:** 1 Department of Research, The INCLEN Trust International, New Delhi, India; 2 Faculty of Management Studies, University of Delhi, New Delhi, India; 3 Faculty of Public Health of Royal College of Physicians, Hon Snr lecturer Imperial College, London, United Kingdom; 4 Centre for Social Medicine and Community Health, Jawaharlal Nehru University, New Delhi, India; 5 Department of Community Medicine, University College of Medical Sciences and Guru Teg Bahadur Hospital, New Delhi, India; 6 Department of Biochemistry, All India Institute of Medical Sciences, New Delhi, India; 7 National Brain Research Center (NBRC), Manesar, Gurgaon, Haryana, India; 8 Department of Paediatrics, Lady Hardinge Medical College, New Delhi, India; 9 Department of Public Health, KLE Academy of Higher Education, Belgaum, Karnataka, India; 10 Department of Medicine, All India Institute of Medical Sciences, New Delhi, India; 11 Department of Training, Ministry of Health and Family Welfare, New Delhi, India; CIFRI: Central Inland Fisheries Research Institute, INDIA

## Abstract

Biomedical research collaborations are to be contextualized in the larger global health agenda which also opens up new information pathways, expands research networks, and brings additional resources. A qualitative inquiry was employed to understand the perceived benefits and challenges of research collaborations by biomedical scientists from India (Global South [GS] country) and the Global North (GN). In-depth interviews were conducted with 47 biomedical scientists from India and 06 from the GN. The data was analyzed using the grounded theory approach. Complementarity of skills and resources, access to funds, improved quality of work, an opportunity to conduct multi-centric studies, development of collaborative networks, better and larger number of publications, mutual learning, opportunity to work with credible researchers, address common interests, leverage interpersonal and trusted relationships and larger societal good were some of the critical factors for eagerness of participants in joint scientific endeavors. However, the challenging aspects of dissent and disagreements were the power imbalance between the collaborators, the development of a trust deficit, and local administrative issues. The challenges reported in the current publication, also echoed in several previous publications can be surmounted and negotiated amicably when the rules of the game, law of the land, sharing of the credits, and interest of the collaborating parties are addressed and agreed up in a fair and just manner before the start of the collaboration. Overall biomedical partnerships are complex collaborations with its challenges, the processes are dynamic and outcomes are emergent. This requires constant and proactive evolution of the preparation, implementation and sustainability of the collaborative efforts be it national or international.

## Background

Scientific collaboration immensely benefits the scope, speed, and quality of knowledge generation, dissemination, and translation. This has to be particularly seen in the context of shared global health challenges when national boundaries get blurred and researchers from across the globe come together to find solutions. This was amply evident during the recent COVID-19 pandemic [1]. Collaboration is also a platform for greater and more effective transfer of knowledge and technology between researchers with different domain expertise and from different geographies [2]. Complex scientific problems necessitate collaborations for the resolution of challenges, innovative ideation, promotion of sustainable development, and not the least improvement of cultural understanding through sharing and complementing capabilities [3]. Continuous efforts have been made to partner and collaborate for health research programs, particularly between High-Income Countries (HIC) and Low- and Middle-Income Countries (LMIC) to address important global health and systems challenges [4]. The functionality and sustainability of partnership depend highly upon the communications, and pre-existing relationships between partners and continuing collaborative funding availability [5]. Clear goals and transparency from the beginning motivate the partners to commit working together and develop relations to achieve common goals [6].

A several-fold rise in the number and scale of collaboration between HIC and LMIC researchers has occurred in the past decades with impressive developments in scientific methods, funding opportunities, enhancing research capacities, and generation of evidence to influence policies that address the questions of both local and global health relevance [5, 7–9]. The advantages of such approaches have also resulted in greater interdependence between researchers, scientists, and policymakers from across regions and countries [10]. Collaborations across geographies can result in highly cited papers with global impact; such collaborations facilitate upgrading of research infrastructure at collaborating sites [11].

Notwithstanding the gains of collaborative research, studies have also highlighted the challenges, disruptions, and potential discord between institutions and individuals during collaborative endeavors [12–14]. Even though the developmental partners and funders promote national and international collaboration, some collaborative initiatives are also criticized for unequal partnership [9]. Many HIC-LMIC collaborations originating from the HICs, involve the LMIC partners more for priority setting, data acquisition, and publications [15], but fail to build their capacity [16, 17]. Literature suggests that challenges to successful research collaborations are often multifactorial and revolve around a lack of common understanding, task sharing, ability to influence decision-making, credit sharing, strategies for dissemination of findings, and commitment to equity and justice among the partners [18–20].

The current study is part of a larger study on leadership framework undertaken with 47 Indian and six international biomedical scientists. The objectives of the study were to document (a) reasons for forging research collaborations; (b) challenges faced in collaborative research; (c) actions taken by researchers in the face of challenging collaborations.

## Methods

### Design

The study was an exploratory cross-sectional design using qualitative methods (in-depth interviews, IDIs) to capture the data conducted between October 2018 and September 2019.

### Selection of participants

A national technical advisory group (TAG) was constituted comprising leading biomedical researchers, research managers, and representatives from developmental partners (N = 41). The TAG members, in consensus, prepared a list of bio-medical scientists from across India following two criteria; (i) involvement in one of the three domains, (basic science, public health, or socio-behavioral research); (ii) each of the scientists perceived to be a research leader in their respective fields based on their research profile (scientific publications, area of research, and reference from TAG). A total of 52 biomedical scientists were purposively identified representing three research themes i.e. basic sciences (n = 19), public health (n = 19), and socio-behavioral (n = 14); under four age categories (<45; 45–55; 55–65 and ≥65 years) to reflect early, mid and established career profiles, and four geographic zones (North, South, West, East, and North-East India; based on their work-life location). Out of the 52 biomedical scientists, five expressed an inability to participate due to a lack of time for face-to-face interviews. The final sample consisted of 47 Indian biomedical scientists based at institutions of repute. The participants were well-known Indian biomedical researchers/scientists and had extensive experience in collaborating with national and international researchers. In addition, six international researchers (two basic science; one socio-behavioral, and three public health themes) from Global North (GN, USA, and Australia) with wide experience in scientific collaboration with Indian scientists were invited to participate (four of these were Indian diaspora, now settled in HICs and two foreign origin researcher).

### Interview schedule

An open-ended IDI guide was designed through a comprehensive review of existing literature and insights provides by the Technical Advisory Group (TAG). The questions in the guide focused on aspects of research collaboration relating to (1) the reasons (technical, scientific, and personal) for collaboration and the benefits obtained therefrom; (2) the constraints and difficulties encountered during the conduct of collaborative research; and (3) the strategies adopted by the scientists to overcome the challenges and steps taken to redefine the collaboration strategy. A separate IDI guide was prepared to interview the bio-medical scientists from GN and focused on the following areas: reasons for deciding to collaborate with Indian partners and institutions, experiences of collaboration, and the challenges experienced in executing collaborative research in India. The IDI guides were refined and finalized after various rounds of pretesting involving three scientists from three premier teaching medical institutions in Delhi; they were not part of the main study participants.

### Data collection

**Fig 1:**
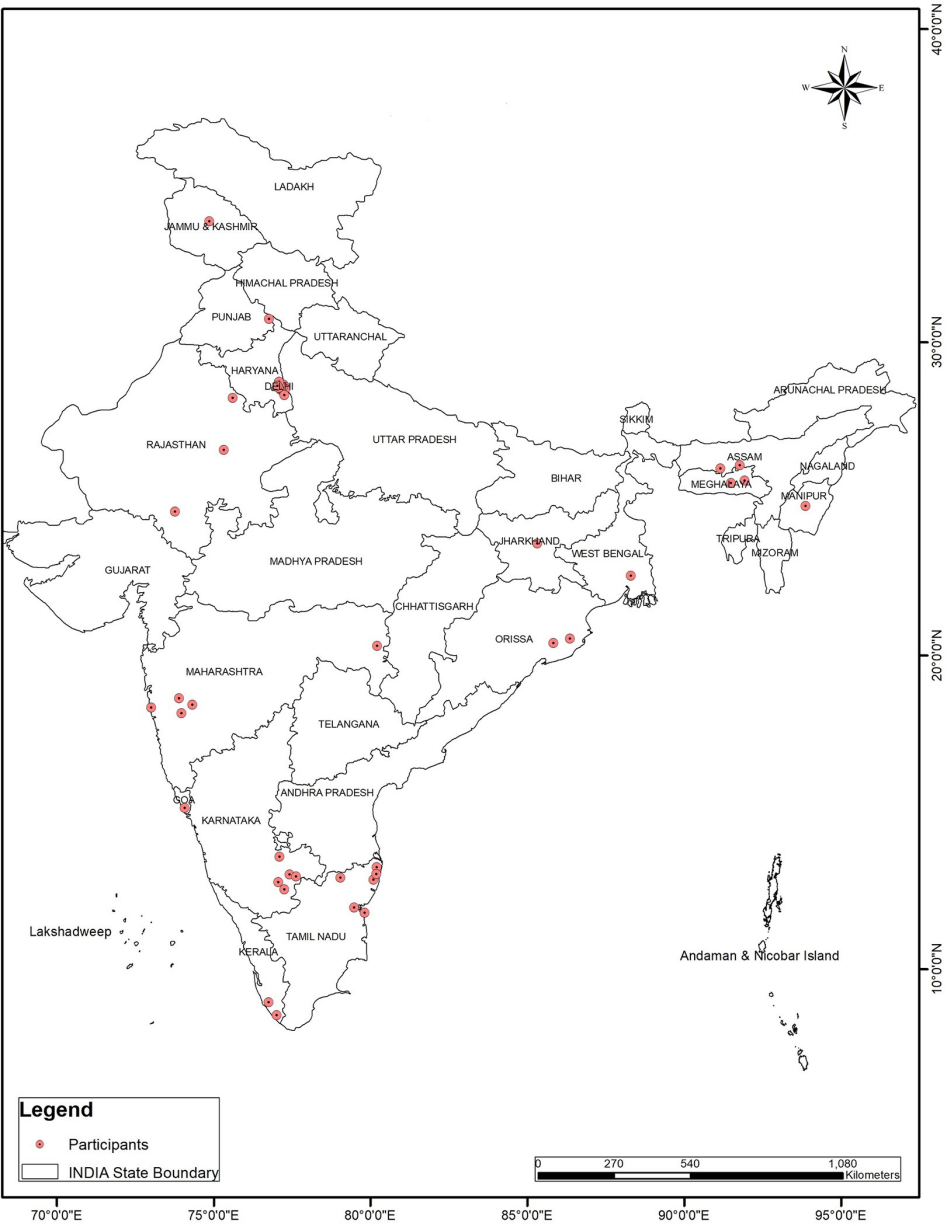
Geographic distribution of index participants (N-47).

Face-to-face IDIs were conducted with 47 Indian researchers from the three research domains (Fig 1). Prior consent and appointment were sought from them for conducting the interviews. The interview was conducted at the place of work of the participants and confidentiality was maintained. The interviewer panel consisted of 40 members (PhD, MD), mid to senior-level public health and/or social scientists, who were researchers/faculty at medical colleges/universities or independent researchers (Male/Female) with experience in qualitative research and IDI conduct. A meeting of all the interviewer panelists was convened for orientation and uniform comprehension of the study protocol, methodology, and IDI guide prior to the data collection. A pair of interviewers thereafter visited the selected participants to conduct the IDIs. The interviews with the international researchers from GN were conducted virtually.

The median duration of the interview with the participants was 119 minutes (range: 59–261 minutes). All the interviews were audio recorded with prior permission and if the interviewer had any comments, these were also recorded at the end of the interview.

Participant validation exercise was conducted with the Technical Advisory Group (TAG) (41 members) and 14 study participants (working in 14 institutions spread across 8 states and 5 zones). The process was completed in three steps. Step 1: We first analysed the qualitative data collected through in-depth interviews of researchers using grounded theory approach and attempted to capture the emerging themes and interpretations. *Step 2*: The synthesized and analyzed data was presented to both the study participants and TAG members to check the trustworthiness and accuracy of the findings. *Step 3*: We organized a one day meeting to seek feedback for interpretative (construct and face) and descriptive validity of the data. This collaborative process provided participants with the opportunity to explore disconfirming voices (objectivism), the ability of the participants to relate these to their personal experiences and the potential addition of data (constructivism) [21–23]. We could not involve researchers from Global North due to logistic reasons.

### Data analysis

The audio recordings of all the interviews were transcribed verbatim by an independent team. The transcribed texts were cross-checked by the study team with the respective audio recording (full duration) to ensure content accuracy. The transcribed data was transferred to INCLEN Qualitative Data Analysis Software (IQDAS, an in-house developed qualitative data entry and management software by INCLEN) for managing data. The entered data were checked for completeness and correctness. We adopted a grounded theory analysis approach to develop an inductively derived explanation of the phenomena emerging from the data [24]. The data analysis team comprised investigators with qualitative research experience (NKA-23 years; VD-15 years; RM-3 years; AM-5 years; JK-1 years). The themes were inductively derived through the iterative process of pile sorting, open coding, axial coding, and selective coding of the data by the research team. To check the inter-coder reliability, the investigators discussed the coding and themes derived from the data and ratified by the senior investigator (NKA). All analytic steps were followed (open, axial, and selective coding) consistently to have inter-coder reliability stability, and reproducibility [25]. The observations were then summarized and interpreted with inputs from the remaining authors. The relevant/important statements or quotes were marked for use as ‘Quotable-Quotes (QQ)’. Data quality was ensured by triangulating data across participant responses. The framework of presenting qualitative responses in a semi-quantitative manner is as follows (values in the parenthesis represent the responder %): very few (< 10%), some (10–24%), about half (25–49%), the Majority (50–75%), most (76–89%), and almost all (>90%). The quasi-quantitative expressions were based on how many times the code was mentioned [26, 27].

We were cognisant of the age and collaborative research experience of the respondent and therefore analysis initially started with taking six from each age group (this was the minimum number of respondents in any age category). After analysing the initial 24 IDIs, no new major themes emerged, indicating data saturation. Thereafter, the complete analysis of 47 IDIs was done and we could get sub-themes like contribute effectively to societal good, science and, collaborators not ready to change.

### Ethical considerations

The study was conducted in compliance with the Indian Council of Medical Research National Ethical Guidelines involving Biomedical and Health Research involving Human Participants (2017). The study protocol (IIEC054) was reviewed and approved by the INCLEN Independent Ethics Committee. Written informed consent was obtained from all the research participants before the interview.

## Results

The profile of the study participants (national and international) is presented in Table 1. Each research participant had experience with at least three or more collaborative works either national or international or both. The participants interviewed from GN had worked in collaboration with Indian institutes and researchers for 20 to 25 years in the fields of demographics, child health, nutrition, and neuroscience.

**Table 1:** Profile of the participants.

Profile of the participants		Biomedical researchers (National)	Biomedical researchers (HIC)
**Themes**	Basic science	18	2
	Socio-behavioral	11	2
	Public health	18	2
**Age in years at interview**	<45	6	
	45–55	8	
	55–65	19	6
	≥65 year	14	
**Location (in India)**	North region	16	
	South region	13	
	West region	7	
	North-East region	11	
**Gender**	Male	36	5
	Female	11	1
**Position/Designation**	Professor and above	33	6
	Associate/Assistant Professor	05	
	Non-teaching researchers	09	

### Findings from client validation exercise

Broadly, the study participants concurred with the data and felt that the study had captured the details related to engagement and collaboration for bio-medical research in the Indian context fairly well. However there were suggestions that point of conflict between collaborators mentioned under the themes: official and scientific stature of the collaborators, hierarchy, professional closure and oppression/dominance fell under the major theme of power struggle during collaboration (Interpretative validity). Importantly as part of the validation exercise, participants re-emphasized the imperatives of the collaborations for their professional growth, scientific advancement and larger societal good, and wanted guidance for approaching national and international research funding sources to consider the findings of the current study for designing the future RFAs.

On the basis of the objectives, the results were analyzed under three broad themes; (a) reasons for forging research collaborations; (b) challenges faced in collaborative research; (c) actions taken in the face of challenging collaborations.

### Reasons for research collaborations

**Fig 2:**
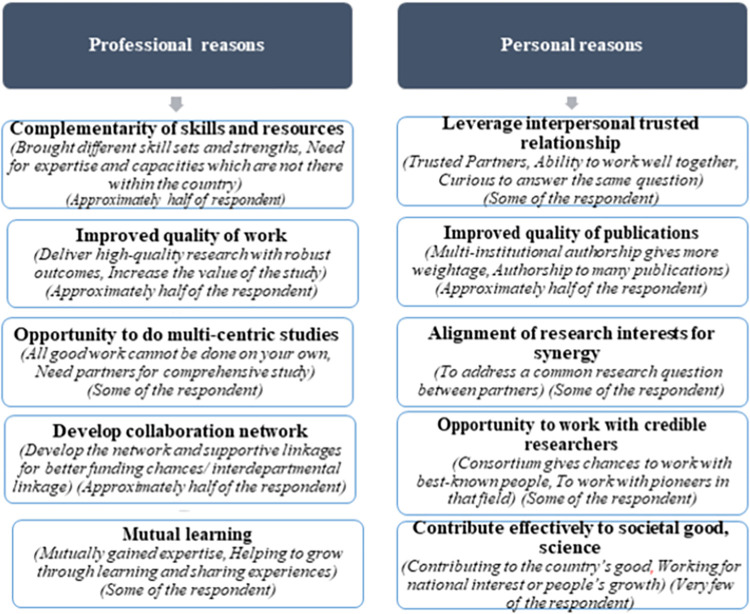
Reasons for pursuing collaboration research.

A range of professional and personal reasons for undertaking collaborative research were identified by the Indian researchers/scientists (Fig 2). The frequently cited reasons for collaboration were: sharpening of the skills, complementarity of capacities and the resources, better quality and impactful scientific work and publications and developing scientific and social network. Contribution to societal good was not perceived to be an important and significant reason for working together.

#### i) Professional reasons for pursuing collaborative research

Indian participant researchers/scientists perceived collaboration as a platform to integrate interdisciplinary and complementary skill sets and to enhance technical and methodological strengths. Each partner in the collaboration brought technical strengths to the research group which in turn resulted in the conceptualization, design, and execution of quality scientific studies and facilitated successful achievement of deliverables. The national and international exposure of researchers from collaborative partnerships brought about an improved work culture and contributed to strengthening the research-friendly environment among researchers and their parent institutions. They *“acquired complimentary professional strengths and got access to latest technology*, *resources and built their infrastructure”*. The value proposition of collaboration perceived by the three categories of Indian researchers different somewhat: the basic scientists leveraged complementary professional strengths in joint projects more as compared to the public health scientists (some) and social scientists (approximately half). Collaborative studies also provided opportunities for mutual learnings, particularly appreciated by the social scientists. Collaborative research was often multi-centric and multi-country, considered generalizable, and applicable in cross-cultural contexts, and findings of collaborative research found a place in the development of relevant national policies and programs. Importantly such studies offered opportunities for networking and found ready support from donors and funding agencies.

“*Science can be impersonal*, *in the sense that you look at the work they have done and work they have published and you attribute it to your collaboration*.*” (R36-Basic Science*, *South India)*

“*Collaboration helps you to break the boundaries*.*” (R7-Social and Behavioral Science*, *North India)*

“*We also look for what the other partner brings to the table*. *We don’t agree to have partners*, *who just have a name*. *-—If you don’t bring working value to the entire group*, *we won’t work with you*. *(R-17-Public Health*, *South India)*

The GN-based researchers held the impression that only a few of the Indian institutions had a commitment to biomedical research and the reputation of these institutions was instrumental in influencing public policy in India with the work being done in these institutions.

#### ii) Personal reasons for pursuing collaborative research

Almost all the participant researchers acknowledged that collaborative studies helped build the technical capacity and skills of both the senior research investigators and their research teams. Most participants were of the opinion that trust in the research relationship and their ability to work together encouraged them to collaborate with their mentors, mentees, friends, and time-tested collaborators. Mutual trust, and transparent communication during the conduct of collaborative research studies with ‘equal’, ‘appreciative’, ‘accommodative, and respectful’ partnerships were important components for sustaining collaboration. Collaborative research resulted in ‘improved quantity and quality of publications’ providing a boost to the participants’ careers. ‘Alignment of research interests’ ‘opportunity to work with credible researcher’ and ‘larger societal good’ were some of the other important reasons for research collaboration. Opportunity to work with well-known investigators was sought for more often by the social scientists compared to other categories. Collaboration occurred and succeeded when mentors believed in ‘treating their former students as equals’.

“*Collaboration is one way of growing*.*” (R-41*, *Social and Behavioral Science*, *West India)*

“*A good successful administrator is that who adapts best practices of other departments or other institutions to your system*.*” (R-33*, *Basic Science*, *South India)*

“*To me*, *that personal trust is extremely important because otherwise*, *it’s always someone watching over our shoulders to see what is happening”*. *(R-17*, *Public Health*, *South India)*

“*It depends on a personal level because you really need to know the person*. *You can’t just go out and collaborate with anyone*. *I know the quality of the work they do and makes me do good quality research*.*” (R-25*, *Public Health*, *North India)*

The respondents from the GN acknowledged having built good professional relationships with their Indian collaborators; they understood and respected each other’s cultural differences. The investigators from GN were enthusiastic about collaborating with Indian scientists due to the prevailing academic standards, the general perception of their hardworking nature and ease of communication, passion for research, integrity, and honesty, adherence to the research protocol, accountability, meticulousness, and data transparency. Past experience, training of Indian scientists in the GN, and their reputation as researchers (quality of publications) in their respective fields were reported to be vital in choosing collaborators. It was not uncommon that the initiative to collaborate came from Indian investigators/ scientists. However, both sides looked for complementarity in personal skills and capacity (Table 2).

“*I look for excellence and complementarity”*. *(R-48*, *Basic Science*, *Global North)*

“*I think what to me is important in collaboration is someone*, *who is really committed to the research” (R-49*, *Public Health*, *Global North)*

“*Not having a critical mass is a big burden…*. *lot depends on the researcher’s excellence and ability to contribute” (R-48*, *Basic Science*, *Global North)*

“*I think getting to know people face-to-face makes it much easier to understand cultural differences and respect one another’s cultural differences*. *So I think it is a big positive*.*” (R-49*, *Public Health*, *Global North)*

**Table 2:** Perception Global North researchers on investigator, institutions, and research in India.

Investigators and research scholars in India	Research institutions in India	Research in India
• Enthusiastic, eager, and interested in good research despite institutional and societal biases or constraints • Hard-working with the knack of acquiring new skills • Dedicated to social good • Helpful to each other • Have the zeal, integrity, and honesty for research • Adheres to protocol and are meticulous in their work • Holds accountability for research • Support data transparency	• Some of the Indian institutions were committed to research • Reputed institutions helped the Global North investigators gain visibility and influence public policy changes • The credibility of institutions increased the probability of collaborative research getting funded • Besides experience, institutions with good knowledge and understanding of collaborative research were perceived as useful	• Collaborative research projects in India have been very productive which led to policy changes and opened further scope of research • Collaborative research in India often has a global impact that leads to the development of global policy • Young researchers with new ideas and approaches have stepped in giving research in India a major boost • The increase in the number of institutions set up for research investigate different areas and avenues of research

### Challenges faced in collaborations in health research

Most of the Indian participants (38 out of a total of 47 participants) admitted to confronting one or more challenges, at least on some occasions, during the conduct of their collaborative research projects. According to the majority of the respondents, the key reasons for conflict and dissent between the collaborators were lack of mutual trust and power imbalance. The third factor with potential for conflict was related to the local administrative challenges (Table 3). The participants resonated that *‘some learning’* always took place with collaborations, and the same was valuable for enriching future partnerships. It was largely believed that *‘good collaborations’* strengthened interpersonal and professional relationships paving the way for future studies. On the other hand, *‘bad collaborations’* left the participants with lessons to redefine future collaboration strategies.

**Table 3:** Challenges faced in collaborations by the index participants.

Themes	Sub-themes	Challenges faced in collaborations
**Trust deficit and lack of confidence**	**Trust-deficit**	• **Unsure relationship** *(Commitment not kept/peer jealousy/collaborator not cooperative/lack of time to support/publication without informing)* (Almost half)
		• **Different scientific interests** *(Compulsive conflict of interest/clash of ideological views/Individual differences in opinion/non-alignment of collaborative goals)* (Some)
**Power struggle between partners**	**Scientific stature of the collaborators**	• **Not a win-win situation among collaborators/ unequal partnership** *(efforts to exert control over the partners/collaboration controlled by a partner/the superiority complex work allocation is heavily loaded towards one side/collaborators try to take more credit than they have contributed)*(Almost Half)
	**Official hierarchy**	• **Agency relationship between mentor-mentee/ collaborators control/collaboration between too junior and too senior** *(a lot of hierarchy and bureaucracy*, *Principal versus rest of investigators)* (Some)
		• **Collaborators not ready to change/ collaborators trying to push unrealistic changes** *(High expectations from low resources; unreasonable timelines; forcing views led to compromised quality)* (Few)
	**Professional closure**	• **Differences in capacity** *(lack of bio-banks; differences in infrastructure and training in terms of statistical and laboratory facilities*, *the difference in competencies*, *lack of protected time for research)* (Some)
	**Oppression**	• **Lack of mutual respect in a relationship** *(Faces humiliation; did not respect partners*, *a derogatory attitude*, *insensitive towards local people)* (Some)
**Administrative and Institutional barrier**	**Administrative**	• **Administrative Hurdles** *(working in the ambit of policies of the government*, *hands are tied*, *roadblocks*, *need to obtain a lot of approvals and consensus; financial entanglements; remote location of the institution)* (Some)

#### 1) Theme 1: Trust deficit and lack of confidence

Trust deficit occurred particularly between the Indian and international investigators when either party breached the *‘rules of the game’*, *i*.*e*. prior agreements. Collaborators also lost trust and confidence in each other when they continued to maintain distinct ideological views and opinions despite working on common research protocols. This led to non-cooperation, and lack of transparency which in turn affected the quality of work. The participants informed that there was a general tendency of some of the GN collaborators to view the Indian partner as a mere ‘*data collector*’, and went ahead with the analysis and publication of data without information, co-authorship, or acknowledgment. Biomedical scientists consistently reported experiencing trust deficits and related conflicts. These issues appeared to be common across their experiences. On the contrary, the scientists from GN perceived a lack of concern for the quality of work, deviations on the agreed upon protocol and a non-committal attitude towards deadlines and/or work promised by some Indian partners as the reasons for conflict. Some GN scientists, recounted instances when the collaboration had to be called off due to protocol violations and poor data monitoring leading to the collection of compromised quality data despite repeated reminders.

“*I learned one thing before I entered into collaboration*, *I would make sure my collaborators had a spine and they would not change like this*.*” (R-30*, *Social and Behavioral Science*, *North India)*

“*It is important that the investigators are able to defend their data and work when questioned*, *as it reflects the integrity of their work*.*” (R-35*, *Basic Science*, *Northeast India)*

“*Irrespective of who you are in the field*, *if you don’t bring value to the entire group*, *we wouldn’t work with you*.*” (R-17*, *Public Health*, *South India)*

“*If uniformity has to exist then the knowledge*, *skill*, *attitude*, *running of the project*, *everything should be as similar as what you are doing” (R-6*, *Basic Science*, *South India)*.

Respondents from GN felt that several Indian investigators were “difficult to work with” due to the unrealistic authorship expectations in publications. Overall international researchers preferred to continue with collaborations where they had greater confidence due to past associations. In general, they did not believe in experimenting or taking risks for new collaborations.

“*I would mostly rely on older collaborators*, *previous collaborators and not develop new relationships*.*” (R-49*, *Public Health*, *Global North)*

“*Usually Indian institutions have a very hard time sharing data*.*” (R-51*, *Social and Behavioral Science*, *Global North)*

“*Doing research in India is hard*, *it is hard anywhere*, *and in India*, *it is doubly hard*, *particularly in leading-edge fields where you see that colleagues in the west or in the north have better resources” [R-48*, *Basic Science*, *Global North)*

“*There is a fairly strong contrarian streak to Indian scientists and academics that sometimes*, *they take a negative tone*.*” (R-49*, *Public Health*, *Global North)*

According to the GN collaborators, Indian researchers were quick to acquire new skills but occasionally demonstrated a lack of willingness as well as the ability to implement or adapt the new skills in their ecosystem. Sometimes the senior leadership in the departments or the institutions of Indian researchers was not receptive to new ideas and thus limited the young researchers from doing creative research.

“*The positive experience for me has been*, *that the work has been productive*, *highly regarded*, *which led to not only further research but policy change and to have a global impact*.*” (R-53*, *Public Health*, *Global North)*

#### ii) Theme 2: Power imbalance between partners

The participants perceived power tussles between the collaborators as the most important reason for conflicts and challenges in continuing with the partnerships. The Indian participants perceived that the international collaborators often demonstrated a condescending attitude since they were often from high-income settings or based in better in-country institutions. In view of the Indian researchers, the feelings of superiority on the part of GN collaborators transformed into efforts to dominate in decision-making, enforcing their views, unequal distribution of workload, discriminatory use of data, uninformed publications, and inequitable credit sharing. Some collaborators tried to dominate LMIC partners by repeatedly reminding them about the resources generated by them to undertake the ‘study’. Incidents were also cited when the collaborators were insensitive towards the cultural and social context of the research personnel, had a derogatory attitude towards the community wherein the research work was being done and collaborators were humiliated and not involved in major discussions and meetings. The participants informed that disrespect from the collaborators made the sustenance of the partnership through the life of the project unsure. Public health and social scientists acknowledged challenges in unequal partnership more often as compared to basic scientists.

“*Collaboration should be to grow capacity rather than control it*.*” (R-41*, *Public Health*, *Western India)*

“*Collaboration is a double-edged sword*, *I would say” (R-38*, *Public health*, *East-north east India)*

“*There’s a dollar value system and there’s a people value system*. *Most often I find conflict between these two value systems*.*” (R-1*, *Public Health*, *Western India)*

“*It should not be that one person is in the driving seat*, *and the other person just waiting to harvest the benefit” (R-29*, *Basic Science*, *North India)*

“*Forcing to accommodate their views/ forced reviewers to be co-authors*. *Confusion-miscommunication-manipulation with the paper” (R-30*, *Social and Behavioral Science*, *North India)*

Some researchers alleged that the ‘*lack of equivalent competence and capacity’* of the Indian researchers, inadequate institutional infrastructure (e.g. absence of bio-banks), and absence of protected time for research were considered professional closures by the collaborators and led to arguments and discords. Researchers from institutions located in remote areas in India complained of a ‘*geographic location disadvantage’* in collaborating in comparison to those located in metro and/or prominent cities and faced biases from both national and international investigators.

We also explored the possibilities of continued collaborative research activities between teachers/mentors and their former students who were now independent researchers and investigators. Some of the index participants were reluctant to collaborate with their mentors or mentees because, of *“changes in the relationship”*, differences in their domains of *“science*”, and fear of being *“overshadowed by each other’s reputation”*. A few mentors were of the opinion it was in the best interest of their former students to be allowed to pursue science independently and explore domains of research beyond their mentors’ specialization.

“*I think when they start off I am still involved for the first couple of years but I like to distance myself from students and especially students in academia very soon*, *so that they are more independent*.*” (R-17*, *Public Health*, *South India)*

#### iii) Theme 3: Administrative and institutional barriers

Administrative and financial obstacles were major impediments to successful research collaboration. In addition to the problems relating to allocation, administration, and management of funds, the research leaders also reported that funding agencies invested little time and resources in a thorough site evaluation which led to subsequent roadblocks and delays in research. The complicated and strenuous process of obtaining a large number of approvals and consensus was reported as an added burden by the Indian researchers. Besides these, frequent changes in the composition of the research team of the collaborators, high expectations with respect to research output from limited resources, and unrealistic timelines were regarded as unfavorable for good collaboration by the Indian researchers.

“*I feel that when it comes to financial collaboration*, *it becomes more complex*. *If technical collaboration*, *is fine*, *they will give us an actor’s honorary advisor or something like that*. *But if it is sharing of finance*, *how much finance would be allocated to them*, *and how much finance to be allocated to us*? *Then the problem starts—“(R-38*, *Public Health*, *East-North East India)*

Hierarchy and the work processes not infrequently controlled by the international partners, and additional constraints of working within the ambit of national science administrative policies in India resulted in unpleasant collaborative experiences. Two basic requirements for international collaboration, with Indian institutions are the need to have Foreign Contribution Regulation Act (FCRA) registration to receive international funds and the project to have the approval of the health ministry’s screening committee (HMSC). While the participants stated that these arrangements safeguarded the country’s interests, the processes, at times delayed and even jeopardized inter-country partnerships for research.

### Actions taken by researchers in the face of challenging collaborations

The Index participants were also asked to share the strategies they adopted when faced with challenging collaborations. Box 1 presents some actions reported to have been undertaken by the index participants when the collaborations ran into rough weather.

**Box 1. Actions taken/proposed by the participants when faced with challenging collaborations (Overlapping actions taken by the participants)**

╶ ***Choosing the partners and entering in to collaboration***
  ○ Alignment of research interest as the primary parameter
╶ ***Setting and implementing clear terms of partnership*:**
  ○ Defining and communicating roles and deliverables clearly with timelines and implementing frameworks for transparency and accountability from the very beginning of the collaboration
  ○ Explicit and clear agreement on sharing of data, samples, publications and credits **(Constitutive)**
╶ ***Discussing and negotiating by setting priorities*:**
  ○ Resolved issues through discussions and compromising only up to one point or negotiating to bring the collaborative partner on board has been another strategy to deal with issues. **(Consensus)**
╶ ***Taking a stand and prioritizing science and the community’s interest*:**
  ○ In situations of insensitivity towards the community, they reported to have taken a stand and been assertive.
  ○ Did not compromise on values such as quality, science, and community prioriti**es (Negotiation)**
╶ ***Enduring and sustaining collaboration till the end of the project*:**
  ○ Limited interactions and accepted each other’s position, became patient, and did not react.
  ○ Withdrew active involvement by delegation of administrative responsibilities and/ or not aggressively managing or promoting projects.
  ○ Even at times gave more credit to the partner than they deserved while lowering expectations just to sustain the collaboration **(Accommodative)**
╶ ***Spreading the word to other stakeholders*:**
  ○ When faced with unequal collaboration or collaborations without integrity, they wrote the issue to the funding agency and also let the other institutes or collaborators know about the unpleasant experienc**e. (Conflict)**
╶ ***Ending or not continuing the collaboration*:**
  ○ In extreme situations, they removed the collaborator, walked away from collaborations, and ended the probabilities of future collaborations.
  ○ They often decided against imposed projects too. **(Conflict)**

## Discussion

This study presents the experiences of collaborative research from 47 Indian and 06 Global North (GN) biomedical researchers and brings forward their perception of the benefits and challenges of collaboration. The researchers perceived collaboration necessary for the generation of high-quality new scientific evidence, opportunity to get recognition, build interpersonal relationships for future collaborations, and for personal and professional growth. Most of such collaborations were multi-centric and inter-disciplinary, between acclaimed national and international institutions and investigators. Notwithstanding these benefits, the majority of the Indian respondents also experienced challenges during collaboration. The power imbalance between the collaborators was perceived as the key reason for conflicts, dissent, and disagreements. Trust deficit occurred particularly during international collaborative endeavors when either party breached prior agreements and or well-accepted principles of partnership. Administrative issues could also affect the collaborations when due diligence for task assignment, fund management, and credit sharing was not done prior to entering into developing articles of the partnership. The investigators from GN had concerns about the commitment of the Indian investigators to rigorously follow the research protocol, methods and quality of the work and hence generally persisted with their time-trusted partners.

We identified 10 factors (scientific and personal reasons) for pursuing research collaboration (Fig 2). Other authors have also acknowledged similar motives for both national and international collaboration [5, 28–30]. Dusdal *et al* emphasized leveraging the talents and expertise of the collaborators, establishing trust, and good work-style fit along with a shared understanding of disciplinary norms, driven by ideas, and available resources [2]. Parker *et al* reported the additional factors related to pursuing interesting science, involvement of effective leader and scientific value/competence of collaborator, emphasis on capacity building, and openness to discussion on everything including disagreements [7]. Rather than working in silos, the notion behind entering into partnerships has been to solve complex scientific problems and promote sustainable development and cultural understanding [31]. Although the possibility of novelty decreases in large collaborative researches but due to their generalizability, impact is high [32]. Cross-cultural and cross-disciplinary experiences of scientific collaboration were acknowledged by study participants as a process of enhancing technical competence and as an evolutionary process for influence on personality, interpersonal relationship building, and refining working patterns. Rorstad *et al* underscored the generational change of role in international research collaboration: as the age advanced, the investigators were more likely to be immersed in alternative forms of international collaborations such as chairing committees, participating in network management, and assuming diverse leadership responsibilities [33]. Overall the study participants accrued a wide spectrum of benefits at personal, professional, institutional, and societal levels.

Mutual trust has been consistently mentioned as a critical factor in any scientific collaboration. As opposed to the semi-colonial model of unidirectional distribution of knowledge, competence and resources, sustainable collaboration models emphasize the imperatives of mutual trust, mutual capacity strengthening, and shared decision-making (bi-directional) [34]. Several studies emphasize the value of trust and confidence among partners as powerful justification for sustainability and durability of research collaborations [7, 35–37]. Trust and confidence hinges on equity, fairness, transparency and adherence to the terms of pre-collaboration agreements. Faure *et al* identified exclusion of LMIC researcher from the research system values (including recognition, reward, publication and grant funding), prioritization of donor interest and LMIC institutions recognized as a sub awardees for funding as important reasons of inequity and fairness in collaborative endeavors [38]. The data or sample sharing with the potential collaborators depends on the national guidelines, and agreement done at the time of initiating collaborations but interpersonal trust and previous experiences linger on as paramount factors for the quality of collaboration [7]. Based on the experiences during COVID-19 pandemic, Modlin *et al* in their recent publication have reiterated the critical need to focus attention on the multiple dimensions of partnership addressing equity and taking steps to prevent hierarchical structures that raise issues of trust and confidence among partners [39]. Careful and comprehensive development of work agreements, adherence to pre-launch agreements and maintenance of a just and fair approach to resolving all concerns were seen as important strategies for gaining and maintaining trust for both present and future collaborations [40, 41].

Our study also highlighted that power tussle between collaborators was one of the central reasons for not continuing with long-term relationships and is consistent with the other studies. This concern of persisting power imbalance has been voiced by LMIC-based researchers, where often they perceived themselves as a ‘data collector/glorified field worker’ i.e. researcher is responsible for providing data, but being excluded from the scientific, creative tasks and talks of the research and fair credit sharing [14, 42, 43]. The power imbalance and collaborative inequities are reported not only in international but also intra-country collaborations [44, 45]. An important factor associated with power imbalances arises from the control and dependency on technical and financial resources. Walsh *et al* reported the handling of funds as important factor for mistrust between collaborators; this often occurred when the bulk of the funding originated in HIC and was generated by the northern partners [46]. According to social exchange theory, the notion of reciprocity and the exchange of resources is critical to sharing benefits and obligations among collaborators [47, 48]. Perceptions about lack of equity and fairness further amplify power imbalances and trust deficits. [9]. Gaillard *et al* encountered problems in the implementation of collaborative research programs due to the asymmetry of distribution of resources and the dominance of the partners in the North [9]. The researchers and science management in developing countries generally pay little attention to ownership, sustainability, and development of national research agenda and capacity development, and therefore often get driven by investigators from the North [34, 49]. Rose *et al*. in their recent publication on one hand recognized the imperatives of strengthening scholarly identity and overcoming funding and mentorship challenges of LMIC researchers in collaborative endeavors but at the same time they experienced research colonialism [50]. The collaborative studies during the COVID 19 pandemic yet again brought forward existing asymmetries; the design, funding and leadership tended to be retained mostly in HIC settings and missing key LMIC-specific input [51, 52].

The collaborators from the GN who participated in the study also had their important perceptions and concerns about Indian researchers. Uniformly they viewed Indian investigators as enthusiastic and interested in good research despite institutional, administrative, and societal constraints, held accountability for research, and supported data transparency. However, they felt that Indian researchers were hesitant to adopt and implement new methods, skills and limited desire to ‘change’ their research ecosystem. The GN researchers also expressed rising concern about ethics and willingness to share data by local scientists. Published studies have echoed similar concerns about the unequal sharing and use of data in collaborative research [7]. It appears that the challenges of north-south collaboration continue to persist in some measure over the time [53].

Financial ambiguities, administrative roadblocks, bureaucracy, and management capacity were reported as additional barriers to the smooth conduct of collaborative research; some participants were apprehensive of embarking on collaborative or network studies due to these reasons. Varshney *et al*. describe that administrative challenges are usually related to limited or no communication between the partners possibly due to differences in the education system, language, and culture of the institution [54].

The landscape of collaborative research is rapidly evolving, and this dynamism may lead to changes in near future. Over time research collaborations between and north and south have increased but several of the challenges reported in our study persist in variable forms [55]. Mutually beneficial North-South collaboration is not an impossible task [56]. One of the key principles of complex collaborations is to identify points/areas of interdependency in these partnerships [57]. The increase in collaborative health research particularly the international collaborations during the last several decades witnessed developments in the scientific methods, trans-sectoral approaches, building of technical capacities and skills in LMICs, innovative funding mechanisms, and policies to jointly address the scientific questions on global health problems and the implementation of programs [58]. In fact, the recent COVID-19 pandemic has amply demonstrated the criticality of such collaboration for the benefit of all communities globally [1, 59]. For addressing the diverse forms of hitches in scientific collaboration, the researchers adopted several strategies outlined in BOX 1. The key strategies recommended by the researchers were better alignment of the research interests between partners and well-structured carefully drawn pre-collaboration agreements on all aspects including funding, common grounds of benefits between partners, sharing of the data and credits. Similar framework developed based on theory of partnerships and empirical evidences has been suggested by Meißner *et al*. [60]. To address power struggle arising out of the access to funds and the funding sources, in recent years, Indian science agencies have started bilateral research programs with the funding for the Indian and collaborating country components comes from respective funding agencies [61].

Limitations: The findings of our study may not directly be applicable to other fields of sciences due to distinctive context and characteristics of biomedical research collaborations. However, we feel that several of the fundamental principles of international partnerships and working together, benefits, and challenges of collaboration might be applicable in different domains of scientific enquiry [62]. One of the limitations of the study was the inclusion of only six international/Global North (GN) researchers who had extensive prior experience partnering with Indian investigators; five of these were from the USA and one from Australia. The views of collaborators from the USA and Australia enriched and augmented the value of the findings of this study although obtaining the perspectives of more investigators spread over several high-income countries would have been desirable. Data indicate that the USA, Germany, China, England are the top biomedical collaborative partner for India [63]. Over two third of the respondents were above the age of the 55 years. We have been cognizant of the age and collaborative research experience of the respondents. We systematically analyzed and compared the perceptions those above and below 55 years but did not find differences in their experiences. There is no central database of both international and national collaborations about biosciences available in India to determine their outcomes. The participants of the study were among the more successful researchers in the country with extensive international collaboration. Although the data is qualitative and reflects the personal experiences and perspectives of the participants, the findings resonate well with published studies from across the HIC and LMICs in a consistent manner. Furthermore, the research collaborations are complex and explaining them in quantitative terms will be challenging [57].

In conclusion, scientific collaborations were considered essential for the advancement of science and solving complex global health problems. Biomedical partnerships are complex collaborations with inherent challenges, the processes are dynamic and outcomes emergent. This requires constant and proactive efforts for nurturing and sustaining the collaboration [64]. The challenges reported in the current publication, also echoed in several previous publications can be surmounted and negotiated amicably when the rules of the game, law of the land, sharing of the credits, and interest of the collaborating parties are addressed and agreed up in a fair and just manner before the start of the collaboration. The benefits of collaborations are multi-directional, multi-sectoral and win-win for all the partners including the larger global community.

## Supporting information

S1 Table Respondent codes. (PDF)

S2 Table COREQ checklist. (PDF)

S3 Table Reasons for collaboration. (PDF)

S4 Table Challenges in collaboration. (PDF)

## References

[1] Druedahl LC, Minssen T, Price WN. Collaboration in times of crisis: A study on COVID-19 vaccine R&D partnerships. Vaccine. 2021; 39:6291–6295. 34556366 10.1016/j.vaccine.2021.08.101 PMC8410639

[2] Dusdal J, Powell JW. Benefits, Motivations, and Challenges of International Collaborative Research: A Sociology of Science Case Study. *Science and Public Policy*. 2021; 235–245.

[3] Sonnenwald DH. Scientific collaboration. Annual review of information science and technology. 2007; 41: 643–681.

[4] Olenko X, Pager S, Holden L. A thematic analysis of the role of the organization in building allied health research capacity: a senior managers’ perspective. BMC Health Service Research. 2012; 12, 276. 10.1186/1472-6963-12-276 PMC3464180 22920443

[5] Dean L, Njelesani J, Smith H, Bates I. Promoting sustainable research partnerships: a mixed-method evaluation of a United Kingdom–Africa capacity strengthening award scheme. Health Research Policy and Systems. 2015; 13, 81 doi: 10.1186/s12961-015-0071-2 26695073 PMC4689047

[6] Iglič H, Doreian P, Kronegger L, Ferligoj. With whom do researchers collaborate and why?. Scientometrics. 2017; 112: 153–174. doi: 10.1007/s11192-017-2386-y 28725095 PMC5486904

[7] Parker M, Kingori P. Good and Bad Research Collaborations: Researchers’ Views on Science and Ethics in Global Health Research. PLoS ONE. 2016; 13: 11(10):e0163579. doi: 10.1371/journal.pone.0163579 27737006 PMC5063577

[8] Lasker RD, Weiss ES, Miller R. Partnership Synergy: A Practical Framework for Studying and Strengthening the Collaborative Advantage. Milbank Quarterly. 2001; 79: 179–205 doi: 10.1111/1468-0009.00203 11439464 PMC2751192

[9] Gaillard JF. North-South research partnership: Is collaboration possible between unequal partners?. Knowledge and Policy. 1994; 7: 31–63.

[10] Opportunities, challenges, and good practices in international research cooperation between developed and developing countries. OECD Global Science Forum, 2011

[11] Csomós G, Vida ZV, Lengyel B. Exploring the changing geographical pattern of international scientific collaborations through the prism of cities. PloS ONE. 2020;15:e0242468. doi: 10.1371/journal.pone.0242468 33196668 PMC7668612

[12] Hedt-Gauthier B, Airhihenbuwa CO, Bawah AA, Burke KS, Cherian T, Connelly MT, et al. Academic promotion policies and equity in global health collaborations. The Lancet. 2018; 392: 1607–1609. doi: 10.1016/S0140-6736(18)32345-6 30496066

[13] Ager A, Zarowsky C. Balancing the personal, local, institutional, and global: multiple case study and multidimensional scaling analysis of African experiences in addressing complexity and political economy in health research capacity strengthening. Health Research Policy System. 2015; 13, 5. doi: 10.1186/1478-4505-13-5 25595847 PMC4325946

[14] Matenga TFL, Zulu JM, Corbin JH, Mweemba O. Contemporary issues in north–south health research partnerships: perspectives of health research stakeholders in Zambia. Health Research Policy System. 2019; 15–17. doi: 10.1186/s12961-018-0409-7 30646902 PMC6334387

[15] Basu A, Kumar BSV. International Collaboration in Indian Scientific Papers. Scientometrics. 2000; 48: 381–402.

[16] Barbara C, Parpart JL. Academic-community collaboration, gender research, and development: pitfalls and possibilities. *Development* in *Practice*. 2006; 16: 15–26.

[17] Smith E, Hunt M, Master Z. Authorship ethics in global health research partnerships between researchers from low or middle-income countries and high-income countries. BMC Medical Ethics. 2014; 15–42. 24885855 10.1186/1472-6939-15-42 PMC4061921

[18] Barrett A, Crossley M, Dachi H. International partnerships, collaboration and capacity building in educational research: the EdQual experience. Comparative Education. 2011; 47: 25–43.

[19] Bansal S, Mahendiratta S, Kumar S, Sarma P, Prakash A, Medhi B. Collaborative research in the modern era: Need and challenges. 2019. Indian Journal Pharmacology. 2019; 51: 137–139. 10.4103/ijp.IJP_394_19 PMC6644188 31391680

[20] Gagliardi AR, Webster F, Brouwers MC, Baxter NN, Finelli A, Gallinger S. How does context influence collaborative decision-making for health services planning, delivery, and evaluation?. BMC Health Service Research. 2014; 14: 545. doi: 10.1186/s12913-014-0545-x 25407487 PMC4239386

[21] Torrance H. Triangulation, Respondent Validation, and Democratic Participation in Mixed Methods Research. Journal of Mixed Methods Research, 2012; 6: 111–123.

[22] Creswell J. Educational Research: Planning, Conducting, and Evaluating Quantitative and Qualitative Research. Upper Saddle River: Merrill Prentice Hall, 2002

[23] Birt L, Scott S, Cavers D, Campbell C, Walter F. Member Checking: A Tool to Enhance Trustworthiness or Merely a Nod to Validation? Qualitative Health Research. 2016; 26:1802–1811. doi: 10.1177/1049732316654870 27340178

[24] Strauss A, Corbin J. Basics of qualitative research: Grounded theory procedures and technique, 2nd Edition. 1998, SAGE Publications, Newbury Park, London. ISBN 9780803959408.

[25] Glaser BG, Strauss AL. The Discovery of Grounded Theory: Strategies for Qualitative Research. Chicago: 1996. Aldine Publishing Company.

[26] Dasgupta R, Chaturvedi S, Adhish SV, Ganguly K, Rai S, Sushant L, et al. Social determinants and polio ‘Endgame’: a qualitative study in high-risk districts of India. Indian Pediatrics. 2008; 45: 357–365. 18693373

[27] Das MK, Arora NK, Chellani HK, Debata PK, Meena KR, Rasaily R, et al. Perceptions of the parents of deceased children and of healthcare providers about end-of-life communication and breaking bad news at a tertiary care public hospital in India: A qualitative exploratory study. PLoS ONE, 2021; 16: e0248661 doi: 10.1371/journal.pone.0248661 33735296 PMC7971872

[28] Denis JL, Lomas J. Convergent evolution: the academic and policy roots of collaborative research. Journal Health Service Research Policy. 2003; 2:1–6. doi: 10.1258/135581903322405108 14596741

[29] Miranda JJ, Bernabé-Ortiz A, Diez-Canseco F, Malaga G, Cardenas MK, Carrillo et al. Towards sustainable partnerships in global health: the case of the CRONICAS Centre of Excellence in Chronic Diseases in Peru. Global Health, 2016; 12–29. 27255370 10.1186/s12992-016-0170-z PMC4890274

[30] Karam M, Brault I, Van Durme T, Macq J. Comparing inter-professional and inter-organizational collaboration in healthcare: A systematic review of the qualitative research. 2018. International Journal Nursing Studies. 2018; 79: 70–83. 10.1016/j.ijnurstu.2017.11.002 29202313

[31] Ghosh J, Kshitij A, Kadyan S. Functional information characteristics of large-scale research collaboration: network measures and implications. Scientometrics, Springer, 2015; 102: 1207–1239

[32] Shin H, Kim K, Kogler DF. Scientific collaboration, research funding, and novelty in scientific knowledge. PLoS ONE. 2022; 17: e0271678. doi: 10.1371/journal.pone.0271678 35877773 PMC9312390

[33] Rørstad K, Aksnes DW, Piro FN (2021) Generational differences in international research collaboration: A bibliometric study of Norwegian University staff. PLoS ONE. 2021; 16: e0260239. 10.1371/journal.pone.0260239 PMC8629247 34843540

[34] Costello A, Zumla A. Moving to research partnerships in developing countries. Biomedical Journal 2000; 321: 827–829. doi: 10.1136/bmj.321.7264.827 11009530 PMC1118627

[35] Vangen S, Huxham C. Nurturing collaborative relations: building trust in inter-organizational collaborations. Journal Applied Behavior Science. 2003; 39: 5–31.

[36] Stokols D, Misra S, Moder RP, Hall KL, Taylor BK. The ecology of team science: understanding contextual influences on trans-disciplinary collaboration. 2008. American Journal Preventive Medicine. 2008; 35: S96–114. 10.1016/j.amepre.2008.05.003 18619410

[37] Wilson T, Berwick DM, Cleary PD. What do collaborative improvement projects do? Experience from seven countries. Joint Commission Journal on Quality and Patient Safety. 2003; 29: 85–93. doi: 10.1016/s1549-3741(03)29011-0 12616923

[38] Faure MC, Munung NS, Ntusi NAB, Pratt B, de Vries J. Considering equity in global health collaborations: A qualitative study on experiences of equity. PLoS ONE. 2021; 16: e0258286. doi: 10.1371/journal.pone.0258286 34618864 PMC8496851

[39] Modlin C, Sugarman J, Chongwe G, Kass N, Nazziwa W, Tegli J, et al. Towards achieving transnational research partnership equity: lessons from implementing adaptive platform trials in low- and middle-income countries. Wellcome Open Res 2023, 8:120 doi: 10.12688/wellcomeopenres.18915.2 38089903 PMC10714106

[40] Heather Getha-Taylor, Misty J G, Kempf RJ, O’Leary RO. Collaborating in the Absence of Trust? What Collaborative Governance Theory and Practice Can Learn From the Literature of Conflict Resolution, Psychology, and Law? American Review of Public Administration. 2019; 49: 51–64

[41] Jones J, Barry MM. Exploring the relationship between synergy and partnership functioning factors in health promotion partnerships. Health Promotion International. 2011; 26: 408–420. doi: 10.1093/heapro/dar002 21330307

[42] Jentsch B. Making Southern realities Count Research agendas and Design in North-South collaborations. International Journal Social Research Methodology. 2004; 7: 259–269.

[43] Canario Guzmán J, Espinal R., Báez J, Melgen R, Rosario P, Mendoza E. Ethical challenges for international collaborative research partnerships in the context of the Zika outbreak in the Dominican Republic: a qualitative case study. 2017. Health Research Policy System. 2017; 15, 82. doi: 10.1186/s12961-017-0246-0 28946911 PMC5613490

[44] Ngwenya S, Boshoff N. Self-interestedness in Research Collaboration and its Association with Career Stage and Nature of Collaboration: A Survey of Zimbabwean Researchers. Journal of Empirical Research Human Research Ethics. 2023; 18:189–207. 10.1177/15562646231192808 PMC10496421 37528585

[45] Sugimoto C. R., Larivière V. Measuring research: What everyone needs to know. Oxford University Press. 2018.

[46] Walsh A, Brugha R, Byrne E The way the country has been carved up by researchers: ethics and power in north–south public health research. International Journal Equity Health. 2016; 15, 204. doi: 10.1186/s12939-016-0488-4 27955670 PMC5153695

[47] Ahmad R, Nawaz MR, Ishaq MI, Khan MM, Ashraf HA. Social exchange theory: Systematic review and future directions. Frontiers Psychology. 2023; 13:1015921. doi: 10.3389/fpsyg.2022.1015921 36710813 PMC9878386

[48] Malmström MM, Johansson J. Social exchange in collaborative innovation: maker or breaker. Journal Innovation Enterprises. 2015; 5, 4.

[49] Morton B, Vercueil A, Masekela R, Heinz E, Reimer L, Saleh S, et al. Consensus statement on measures to promote equitable authorship in the publication of research from international partnerships. Anesthesia. 2022; 77: 264–276. doi: 10.1111/anae.15597 34647323 PMC9293237

[50] Rose ES, Bello-Manga H, Boafor T and Asaduzzaman M. International collaborative research, systems leadership and education: reflections from academic biomedical researchers in Africa. Frontiers Education. 2014; 8:1217066. 10.3389/feduc.2023.1217066 PMC13309179 42369580

[51] Adebisi YA. Decolonizing Epidemiological Research: A Critical Perspective. Avicenna Journal Medicine. 2023; 13:68–76. doi: 10.1055/s-0043-1769088 37435557 PMC10332938

[52] Binagwaho A, Allotey P, Sangano E, Ekström AM, Martin K. A call to action to reform academic global health partnerships. BMJ. 2021; 375: n2658. doi: 10.1136/bmj.n2658 34725093

[53] Bratt S, Langalia M and Nanoti A. North-south scientific collaborations on research datasets: a longitudinal analysis of the division of labor on genomic datasets (1992–2021). Frontiers. Big Data, 2023, 6:1054655. doi: 10.3389/fdata.2023.1054655 37397623 PMC10311002

[54] Varshney D, Atkins S, Das A, Diwan V. Understanding collaboration in a multinational research capacity-building partnership: a qualitative study. Health Research Policy and Systems. 2016; 14:64 doi: 10.1186/s12961-016-0132-1 27538447 PMC4991081

[55] Dusdal Jennifer, Justin J W Powell, Benefits, Motivations, and Challenges of International Collaborative Research: A Sociology of Science Case Study, Science and Public Policy, 2021; 48: 235–245

[56] Halstead SB, Tugwell P, Bennett K. The international clinical epidemiology network (INCLEN): a progress report. Journal Clinical Epidemiology. 1991; 44: 579–589 doi: 10.1016/0895-4356(91)90222-u 2037863

[57] Mankin D, Cohen S, Fitzgerald S P. Developing complex collaborations: basic principles to guide design and implementation. Complex Collaboration: Building the Capabilities for Working Across Boundaries, 2015; 1–26

[58] Ihekweazu C, Ncube F, Schoub B, Blumberg L, Ruggles Salter M, et al. A North/South collaboration between two national public health institutes–A model for global health protection. Journal of Public Health Policy. 2015; 36: 181–193. doi: 10.1057/jphp.2014.52 25568964 PMC7100317

[59] Bump JB, Friberg P, Harper DR. International collaboration and covid-19: What are we doing and where are we going?. Biomedical Journal. 2021; 28:372:n180. doi: 10.1136/bmj.n180 33509953 PMC7842258

[60] Meißner F, Weinmann C and Vowe G. Understanding and Addressing Problems in Research Collaboration: A Qualitative Interview Study from a Self-Governance Perspective. Frontiers in Research Metrics and Analytics. 2022; 6:778176. doi: 10.3389/frma.2021.778176 35224422 PMC8864336

[61] India- US Collaborative Vision Research Program Funding Opportunity Announcement. Department of Biotechnology & NEI-NIH. 2023. https://dbtindia.gov.in/sites/default/files/DBT-NIH%20Indo-US%20FoA%20on%20Vision%20Research.pdf. Accessed on 25Jan 2024.

[62] Baker MR, Steins NA, Pastoors MA, Neuenfeldt S, de Boer A, Haasnoot D, et al. A new era for science-industry research collaboration-a view towards the future. Frontiers in Marine Science, 2023; 10:1144181

[63] Rensburg I, Motala S, David SA. Research collaboration among emerging economies: Policy and economic implications for BRICS nations. 2016. International Journal of Economic Policy in Emerging Economies. 2016; 344–360.

[64] Lorange P. Complex collaboration: The case of a business school and its complex network of relationship. Building the capabilities for working across boundaries. 2015; 109–124.

