# Supplementary material for: Challenges of biomedical research collaboration in India: Perceptions of Indian and international researchers
Source: PLoS One. 2024 Jun 28;19(6):e0305159. doi: 10.1371/journal.pone.0305159 (PMC11213314; doi:10.1371/journal.pone.0305159)
Supplement: S1 Table — (PDF) [file pone.0305159.s001.pdf]

Supplementary Table 1: Respondent codes			
S.no (alphabetical)	Discipline	Region (India)	Respondent Code
R1	Public health	West	R1, Public Health, West India
R2	Public health	West	R2, Public Health, West India
R3	Public health	South	R3, Public Health, South India
R4	Basic Science	East-Northeast	R4, Basic Science, Northeast
R5	Basic Science	North	R5, Basic Science, North India
R6	Basic Science	South	R6, Basic Science, South India
R7	Socio-behavioral	North	R7, Social Behavioural Science, North India
R8	Basic Science	West	R8, Basic Science, West India
R9	Basic Science	East-Northeast	R9, Basic Science, Northeast India
R10	Public health	East-Northeast	R10, Public Health, Northeast India
R11	Public health	North	R11, Public Health, North India
R12	Basic Science	West	R12, Basic Science, West India
R13	Public health	East-Northeast	R13, Public Health, Northeast India
R14	Basic Science	South	R14, Basic Science, South India
R15	Basic Science	South	R15, Basic Science, South India
R16	Basic Science	North	R16, Basic Science, North India
R17	Public health	South	R17, Public Health, South India
R18	Socio-Behavioural	East-Northeast	R18, Social Behavioural Science, Northeast India
R19	Public health	South	R19, Public Health, South India
R20	Public health	North	R20, Public Health, North India
R21	Public health	North	R21, Public Health, North India
R22	Public health	South	R22, Public Health, South India
R23	Public health	North	R23, Public Health, North India
R24	Basic Science	North	R24, Basic Science, North India
R25	Public health	North	R25, Public Health, North India
R26	Socio-Behavioural	East-Northeast	R26, Social Behavioural Science, Northeast India
R27	Public health	North	R27, Public Health, North India
R28	Public health	North	R28, Public Health, North India
R29	Basic Science	North	R29, Basic Science, North India
R30	Socio-Behavioural	North	R30, Social Behavioural Science, North India
R31	Socio-Behavioural	North	R31, Social Behavioural Science, North India
R32	Socio-Behavioural	South	R32, Social Behavioural Science, South India
R33	Basic Science	South	R33, Basic Science, South India
R34	Public health	North	R34, Public Health, North India
R35	Basic Science	East-Northeast	R35, Basic Science, Northeast India
R36	Basic Science	South	R36, Basic Science, South India
R37	Public health	East-Northeast	R37, Public Health, Northeast India
R38	Public health	East-Northeast	R38, Public Health, Northeast India
R39	Basic Science	East-Northeast	R39, Basic Science, Northeast India
R40	Socio-Behavioural	West	R40, Social Behavioural Science, West India
R41	Socio-Behavioural	West	R41, Social Behavioural Science, West India
R42	Socio-Behavioural	South	R42, Social Behavioural Science, South India
R43	Basic Science	West	R43, Basic Science, West India

S.no (alphabetical)	Discipline	Region (India)	Respondent Code
R44	Basic Science	South	R44, Basic Science, South India
R45	Basic Science	North	R45, Basic Science, North India
R46	Socio-Behavioural	East-Northeast	R46, SBS Social Behavioural Science, Northeast India
R47	Socio-Behavioural	South	R47, Social Behavioural Science, South India
Global North			
R48	Basic Science	Global North	R48, Basic Science, Global North
R49	Public Health	Global North	R49, Public Health, Global North
R50	Public Health	Global North	R50, Public Health, Global North
R51	Socio-Behavioural	Global North	R51, Social Behavioural Science, Global North
R52	Basic Science	Global North	R52, Basic Science, Global North
R53	Public Health	Global North	R53, Public Health, Global North
