# Supplementary material for: Challenges of biomedical research collaboration in India: Perceptions of Indian and international researchers
Source: PLoS One. 2024 Jun 28;19(6):e0305159. doi: 10.1371/journal.pone.0305159 (PMC11213314; doi:10.1371/journal.pone.0305159)
Supplement: S3 Table — (PDF) [file pone.0305159.s003.pdf]

Supplementary Table 3: Benefits of Pursuing Collaborative Research: Perceptions of Basic Science, Public Health and Social Science Researchers

Reasons for pursuing collaboration	Biomedical Scientist from three domains		
**Scientific**	**Basic Science**	**Public Health**	**Social**
• **Complementarity of skills and resources** *(Brought different skill sets and strengths, Need for expertise and capacities which are not there within the country)*	Most	Some	Approximately half
• **Improved quality of work** *(Deliver high-quality research with robust outcomes, Increase the value of the study)*	Approximately half	Approximately half	Majority
• **Opportunity to do multi-centric studies** *(All good work cannot be done on your own, Need partners for comprehensive study)*	Approximately half	Approximately half	Some
• **Develop collaboration network** *(Develop the network and supportive linkages for better funding chances/ interdepartmental linkage)*	Approximately half	Approximately half	Some
• **Mutual learning** *(Mutually gained expertise, helping to grow through learning and sharing experiences)*	Approximately half	Some	Almost all
**Personal**			
• **Leverage interpersonal trusted relationship** *(Trusted Partners, Ability to work well together, Curious to answer the same question)*	Some	Very few	Some
• **Improved quality of publications** *(Multi-institutional authorship gives more weightage, Authorship to many publications)*	Approximately half	Majority	Approximately half
• **Alignment of research interests for synergy** *(To address a common research question between partners)*	Some	Some	Very few
• **Opportunity to work with credible researchers** *(Consortium gives chances to work with best-known people, To work with pioneers in that field)*	Some	Some	Approximately half
• **Contribute effectively to societal good, science** *(Contributing to the country's good, Working for national interest or people's growth)*	Very few	Very few	-
