# Supplementary material for: Challenges of biomedical research collaboration in India: Perceptions of Indian and international researchers
Source: PLoS One. 2024 Jun 28;19(6):e0305159. doi: 10.1371/journal.pone.0305159 (PMC11213314; doi:10.1371/journal.pone.0305159)
Supplement: S4 Table — (PDF) [file pone.0305159.s004.pdf]

**Supplementary Table 4: Challenges of Research Collaboration: Perceptions of Basic Science, Public Health and Social Science Researchers**					
**Themes**	**Sub-themes**	**Challenges faced in collaborations**	**Basic**	**Public Health**	**Socio-behavioral**
**Trust deficit and lack of confidence**	**Trust-deficit**	• **Unsure relationship** *(Commitment not kept/peer jealousy/collaborator not cooperative/lack of time to support/publication without informing)* (Almost half)	Approximately Half	Some	Approximately Half
		• **Different scientific interests** *(Compulsive conflict of interest/clash of ideological views/Individual differences in opinion/non-alignment of collaborative goals)* (Some)	Very few	Approximately Half	Some
**Power struggle between partners**	**Scientific stature of the collaborators**	• **Not a win-win situation among collaborators/ unequal partnership** *(efforts to exert control over the partners/collaboration controlled by a partner/the superiority complex work allocation is heavily loaded towards one side/collaborators try to take more credit than they have contributed)*(Almost Half)	Some	Approximately Half	Approximately Half
	**Official hierarchy**	• **Agency relationship between mentor-mentee/ collaborators control/collaboration between too junior and too senior** *(a lot of hierarchy and bureaucracy, Principal versus rest of investigators)* (Some)	Some	Some	Very few
		• **Collaborators not ready to change/ collaborators trying to push unrealistic changes** *(High expectations from low resources; unreasonable timelines; forcing views led to compromised quality)* (Few)	-	Some	Very few
	**Professional closure**	• **Differences in capacity** *(lack of bio-banks; differences in infrastructure and training in terms of statistical and laboratory facilities, the difference in competencies, lack of protected time for research)* (Some)	Some	Some	Very few
	**Oppression**	• **Lack of mutual respect in a relationship** *(Faces humiliation; did not respect partners, a derogatory attitude, insensitive towards local people)* (Some)	Some	Some	-
**Administrative and Institutional barrier**	**Administrative**	• **Administrative Hurdles** *(working in the ambit of policies of the government, hands are tied, roadblocks, need to obtain a lot of approvals and consensus; financial entanglements; remote location of the institution)* (Some)	Some	Some	Approximately Half
